# Phylogeny-structured carbohydrate metabolism across microbiomes collected from different units in wastewater treatment process

**DOI:** 10.1186/s13068-015-0348-2

**Published:** 2015-10-22

**Authors:** Yu Xia, Francis Y. L. Chin, Yuanqing Chao, Tong Zhang

**Affiliations:** Environmental Biotechnology Laboratory, The University of Hong Kong, Hong Kong, SAR China; Department of Computer Science, The University of Hong Kong, Hong Kong, SAR China; Department of Computing, Hang Seng Management College, Hong Kong, SAR China; School of Environmental Science and Engineering, Sun Yat-sen University, Guangdong, China

**Keywords:** Metagenomic, Carbohydrate metabolism, Glycoside hydrolase, Temperature, Dissolved oxygen, Salinity

## Abstract

**Background:**

With respect to global priority for bioenergy production from plant biomass, understanding the fundamental genetic associations underlying carbohydrate metabolisms is crucial for the development of effective biorefinery process. Compared with gut microbiome of ruminal animals and wood-feed insects, knowledge on carbohydrate metabolisms of engineered biosystems is limited.

**Results:**

In this study, comparative metagenomics coupled with metabolic network analysis was carried out to study the inter-species cooperation and competition among carbohydrate-active microbes in typical units of wastewater treatment process including activated sludge and anaerobic digestion. For the first time, sludge metagenomes demonstrated rather diverse pool of carbohydrate-active genes (CAGs) comparable to that of rumen microbiota. Overall, the CAG composition correlated strongly with the microbial phylogenetic structure across sludge types. Gene-centric clustering analysis showed the carbohydrate pathways of sludge systems were shaped by different environmental factors, including dissolved oxygen and salinity, and the latter showed more determinative influence of phylogenetic composition. Eventually, the highly clustered co-occurrence network of CAGs and saccharolytic phenotypes, revealed three metabolic modules in which the prevalent populations of *Actinomycetales*, *Clostridiales* and *Thermotogales,* respectively, play significant roles as interaction hubs, while broad negative co-exclusion correlations observed between anaerobic and aerobic microbes, probably implicated roles of niche separation by dissolved oxygen in determining the microbial assembly.

**Conclusions:**

Sludge microbiomes encoding diverse pool of CAGs was another potential source for effective lignocellulosic biomass breakdown. But unlike gut microbiomes in which *Clostridiales*, *Lactobacillales* and *Bacteroidales* play a vital role, the carbohydrate metabolism of sludge systems is built on the inter-species cooperation and competition among *Actinomycetales*, *Clostridiales* and *Thermotogales*.

**Electronic supplementary material:**

The online version of this article (doi:10.1186/s13068-015-0348-2) contains supplementary material, which is available to authorized users.

## Background

Microbial heterotrophic carbohydrate hydrolysis, as a combination of various biochemical processes responsible for the formation, breakdown and transformation of carbohydrates in microorganisms, is the most ancient metabolic pathway whose structure is ultimately determined by the microbial composition in natural environment, host-associated microbiomes and engineering biosystems [1]. Given the global priority for biofuel production from plant biomass (mainly carbohydrates), the metabolic pathways and carbohydrate-active genes from microbial communities in the guts of ruminal animals and wood-feeding insects have been extensively explored for discovery of cellulolytic phenotypes and hydrolytic enzymes [2–4].

On another side, for many years, effective microbial breakdown of oxygen-depleting organic pollutants (mainly carbohydrates) had taken place in wastewater treatment plants (WWTPs) [5]. The hydrolysis of complex organic matter, especially the omnipresent cellulosic component, is the rate-limiting step for anaerobic biofuel generation [6–8]. However, compared with gut microbiota of ruminal animals and wood-feeding insects, the carbohydrate pathways of microbiomes developed in engineered biosystems, like sludge communities of aerobic and anaerobic treatment in WWTPs, remain largely unexplored due to technical bottlenecks including prerequisite of isolation and low-throughput DNA sequencing.

In recent years, our knowledge of microbial metabolisms has been advanced considerably by the technology advance of next-generation sequencing (NGS) techniques. NGS-based metagenomics, studying microbial communities without prior culturing nor marker gene amplification, could provide a relatively unbiased view of not only the community structure (species richness and distribution) but also the metabolic pathways of a community. This promising approach had been successfully use to reveal that the effectiveness of polysaccharides breakdown in human gut which relies heavily on mutualistic cooperation of *Firmicutes* and *Bacteroidetes showing* different affinity to cellulosic substrate with distinctive metabolic mechanisms [1]. Yet, whether such cooperative pattern could be applied or whether alternative microbial interaction exists within microbiota of engineered biosystems requires further investigation.

Here, to fill the knowledge gaps of carbohydrate metabolisms in engineered biosystems, comparative metagenomics based on de novo gene recovery, coupled with community-wide metabolic network reconstruction, was used to examine quantitative distribution and phylogenetic diversity of the carbohydrate-active genes (CAGs) carried by the microbes habitat in different treatment processes of WWTPs. Activated sludge (AS) and anaerobic digestion sludge (ADS) possessing remarkable physiological and functional heterogeneity toward the degradation of carbohydrates, were selected from various treatment units under well-controlled conditions (temperature, dissolved oxygen (DO), and salinity), providing a unique opportunity to explore the impact of operational parameters on carbohydrate pathways within these systems. We present here, the reconstruction of metabolic networks by linking different carbohydrate pathways with microbial groups will extend our knowledge on the inter-species cooperations and competitions in the engineering biosystems.

## Results and discussion

In total twelve sludge metagenomes including nine for comparison and another three as technical or biological replicates (Additional file 1: Figure S1) were sequenced on the Illumina Hiseq 2000 platform with PE101 strategy (three of these metagenomes were included in our previous work for purposes other than studying carbohydrate metabolism [9, 10]). The total metagenomic reads after quality control ranged from 25 to 175 million for sludge microbiomes with increasing diversity (Table 1). Around 0.07 % of the post-QC reads was identified as 16S/18S rRNA gene sequences (Additional file 1: Table S1). On average 51.2 % of the obtained clean reads were included in the de novo assembly (Table 1). Applying gene identification algorithm specifically designed for NGS-based metagenomes, a total of 3,736,227 protein-coding genes were obtained (Table 1). Among them, CAGs comprise roughly 2.8 % across sludge metagenomes (Table 1). Since the dataset size was different among sludge samples, relative abundance normalized against the total number of annotated genes/sequences was used for subsequent statistic comparison.

**Table 1:** Characteristics of sludge samples collected from different processes of wastewater treatment plant

Sample name	Sample collection location	Sample description	Tep	Salt	DO	Post-QC reads number	Reads utilization (%)	ORFs number	CAG-ORFs percentage (%)
Stanley_AS	Stanley WWTP	Suspended proportion of AS-treating fresh wastewater	A	F	Aer	164,298,394	56.4	1,217,440	3.03
Stanley_BF	Stanley WWTP	Attached to the carrier proportion of AS-treating fresh wastewater	A	F	Aer	175,985,866	52.1	1,348,161	1.46
ST_AS_winter	Shatin WWTP	Activated stage treating saline wastewater collected at winter	A	S	Aer	49,496,374	40.6	306,862	1.70
ST_AS_summer	Shatin WWTP	Activated stage treating saline wastewater collected at summer	A	S	Aer	50,164,884	37.4	300,883	1.88
ST_ADS	Shatin WWTP	Full-scale anaerobic digester treating saline wastewater	M	S	An	31,818,174	47.4	158,417	1.86
SWH_ADS	Shek Wu Hui WWTP	Full-scale anaerobic digester treating fresh wastewater	M	F	An	36,670,382	51.5	67,636	6.70
MAD	Lab-scale Anaerobic digester	Lab-scale anaerobic digester at mesophilic condition	M	S	An	25,253,200	42.5	128,509	2.02
TAD	Lab-scale Anaerobic digester	Lab-scale anaerobic digester at thermophilic condition	T	S	An	22,464,032	42.7	116,511	2.25
TCF	Lab-scale Anaerobic digester	Lab-scale thermophilic anaerobic digester enriched with cellulosic substrate	T	F	An	50,255,458	89.8	91,808	4.83

*Tep* category of temperature, *A* ambient temperature, *M* mesophilic, *T* thermophilic, *Salt* category of salinity, *F* fresh, *S* saline, *DO* category of dissolved oxygen, *Aer* aerobic, *An* anaerobic, *Reads utilization* percentage of reads included in the de novo assembly, *AS* activated sludge, *WWTP* wastewater treatment plant, *BF* biofilm, *ADS* anaerobic digestion sludge

Sufficient coverage of the sludge community studied was confirmed by the rarefaction curves of 16S/18S rRNA gene sequences (Additional file 1: Figure S2). Analysis of the two technical replicates of ADS collected at SWH WWTP showed Illumina Hiseq 2000 sequencing had good reproducibility based on the values of slope (approach 1.0) and high linear coefficient (*R*^2^ of 1.0 for both taxa and enzymatic profiles) (Additional file 1: Table S2). The biological replicates of ADS collected at different sampling times showed visible variation in taxa composition (*R*^2^ averagely around 0.8, Additional file 1: Table S2, See Additional file 1: Figure S3 for significantly changed taxa between biological replicates), thus the mean value of biological replicates (when available) were used for statistic comparison among sludge microbiomes. In addition, such variation resembled a reasonable scale for community shift in sludge microbiomes of WWTPs [11]. Noteworthy, stable global functions were maintained even at evident phylogeny variation (*R*^2^ > 0.95, Additional file 1: Table S2), suggesting a reproducible metagenomic quantification of protein-coding genes including CAGs.

### Carbohydrate-active genes of the metagenomic community

Most of the broad array of genes involved in carbohydrate metabolism are GH (glycoside hydrolase) families which hydrolyze the glycosidic bond between carbohydrates or between a carbohydrate and a non-carbohydrate moiety [12]. Besides GH families, the carbohydrate esterases (CEs), catalyzing the deactylation of substituted saccharides, determine the rate of polysaccharides breakdown [13]. In addition, the carbohydrate-binding modules (CBMs) assist in hydrolysis of polysaccharides by bringing the biocatalyst into close contact with its recalcitrant substrate. Therefore, the subsequent discussion will mainly focus on these CAG families.

Based on identification of CAGs’ catalytic domains, sludge metagenomes recorded a diverse profile of carbohydrate pathways of totally 109 GHs, 16 CEs and 64 CBMs families (Additional file 1: Table S3) comparable to those in animal guts. More than half of the GH-encoding ORFs showed less than 60 % similarity to the sequences collected in NCBI *nr* database by BLASTp searching (Additional file 1: Figure S4), indicating the high novelty and our limited understanding of these CAGs’ families retrieved from sludge microbiomes. Alpha-*N*-acetylgalactosaminidase (GH109), multifunctional alpha-amylase (GH13), and endoglucanase (GH74) were the most frequent glycoside hydrolases across sludge habitats implying the common capability of glycoprotein and alpha-/beta- linked polysaccharides breakdown in sludge (Additional file 1: Table S3).

Higher CAG abundance suggested a greater role of carbohydrate metabolism in anaerobic systems (3.5 %) than aerobic ones (2.0 %) (Table 1). This finding was not entirely unexpected, given the predominant content of complex biopolymers such as lipopolysaccharides from dead bacterial cell walls in the anaerobic digestion process of WWTPs. Compared with the gut microbiomes (like bovine rumen [2, 14], termite hindgut [4] and tammar wallaby [3]), comparable abundance of oligosaccharides-degrading enzymes was observed in sludge systems, respectively, 66.0 and 67.2 % for sludge and gut microbiomes, suggesting the importance of cellulosic biomass hydrolysis in sludge (Additional file 1: Table S4). Similar to microbiomes of termite and tammar wallaby, GH5 was numerically most abundant cellulase in aerobic sludge systems with less representation with GH9, while in contrast, the rumen microbiome and anaerobic systems showed a more evenly balanced ratio with respect to these two families (Additional file 1: Table S4).

### Correlation between microbial phylogeny and CAGs

The host taxa of each CAG-encoding ORF was identified using lowest common ancestor algorithm based on its homologies found in NCBI *nr* database. *Actinomycetales* was the most prevalent predicted order in aerobic CAG-active microbes, while *Clostridiales* and *Thermotogales* were the most dominant in anaerobic carbohydrate metabolism (Additional file 1: Figure S5a). Such distribution pattern was in agreement with community phylogeny between overall aerobes and anaerobes based on 16S rRNA genes (Additional file 1: Figure S5b), indicating dominance of CAG-associated microbial populations in sludge microbiomes.

**Fig. 1:**
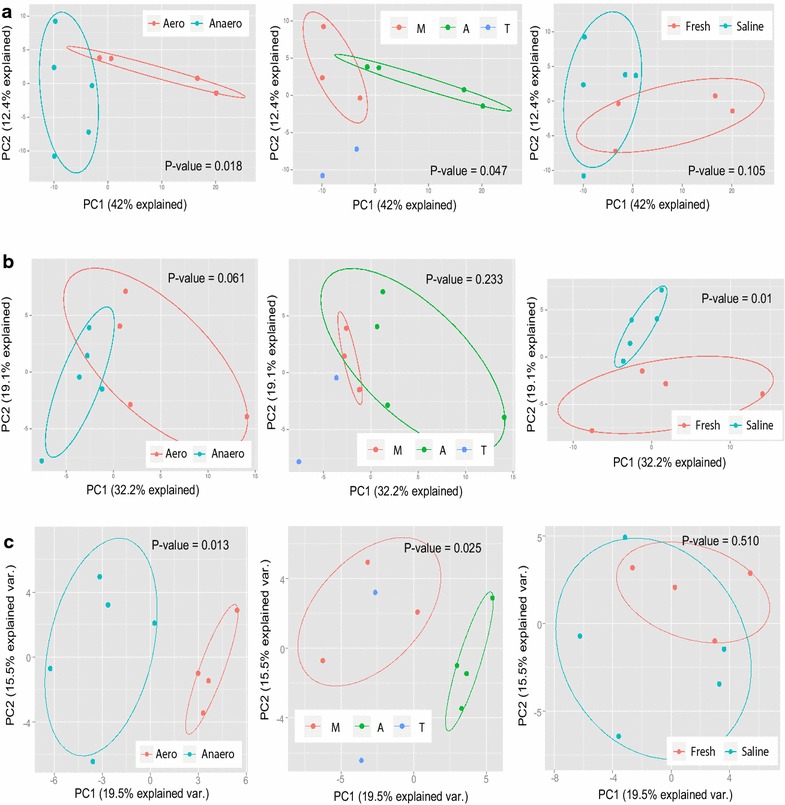
Principal coordinate analysis (PCoA) plots depict Bray–Curtis distance between sludge samples using CAG content (**a**) and phylogeny distribution based on 16S rRNA genes (**b**) or based on CAG-encoding ORFs (**c**). Significance Bray–Curtis distance between groups is indicated by *p* value (analysis of similarity, ANOSIM). *Eclipse* is drawn with confidence limit of 0.68. Samples are, respectively, colored according to environmental categories of DO (anaerobic and aerobic), temperature (*M* mesophilic; *A* ambient temperature; *T* thermophilic) and salinity (saline and fresh) in the *left*, *middle* and *right* subfigures

Taking advantage of the well-controlled conditions (DO, temperature and salinity) in these non-natural engineering biosystems, we investigated influence of environmental factors on the distribution of both microbial phylogeny and CAGs, as well as their correlations. Principal coordinate analysis (PCoA) based on the CAGs’ abundance showed significant partition (defined as *p* value <0.05) of sludge samples with different DO levels (*p* value of 0.02) (Fig. 1a). Compared to aerobic sludge, the significantly prevalent SLH domain (Additional file 1: Figure S6) which could anchor onto the bacterial cell wall polymers suggested recurrent incidence of attachment-based pathways in anaerobic sludge. The phylogeny grouping based on CAG-encoding ORFs also showed a clear separation between aerobic and anaerobic sludge samples (*p* value 0.01) (Fig. 1c), but this clustering boundary became indistinct (*p* value 0.06) when overall community structure was accessed using 16S rRNA gene sequences (Fig. 1b). The occurrence of populations whose prevalence is mainly driven by factors other than dissolved oxygen may be responsible for this variation. Surprisingly, samples with different salinity showed more distinct overall community structure (clustering *p* value of 0.01) (Fig. 1b) implying a more significant impact of salinity on sludge microbial phylogeny. In contrast, only one CAG family, GH13 showed significant shift in abundance along salinity variation (Additional file 1: Figure S6).

**Fig. 2:**
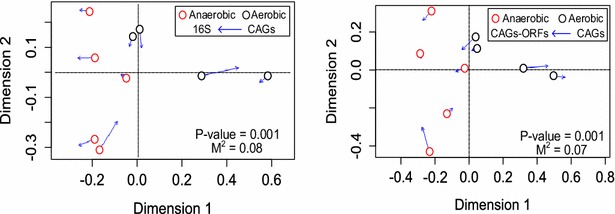
Carbohydrate metabolism correlates with sludge phylogeny across samples with different dissolved oxygen (DO). Procrustes superimposition plot depicts significant (*p* value <0.05) correlation between CAG content (Bray–Curtis) and microbial composition (Bray–Curtis). The microbial composition is determined independently by both 16S rRNA gene sequences (*left*) and CAG-encoding ORFs (*right*). Points representing sludge samples are *colored* according to DO category. Position of points is determined within the dimension after Procrustes transformation. *Arrows* are drawn from the position representing CAG content to that depicting microbial composition. The length of the *arrow line* represents the Procrustes error of the transformation

Correlations between CAG profiles and community compositions were further investigated by Procrustes analyses. Shapes (principle coordinates in this case) showing minimal value of Procrustes distance and fitness measures after transformation indicates strong correlation between observations. Bray–Curtis distance calculated from normalized CAG counts significantly correlated with microbial order inferred from both 16S rRNA sequences and CAG-encoding genes (Fig. 2). Visualized by Procrustes analyses, both the CAG content and microbial composition of all sludge samples consistently displayed highly significant goodness-of-fit measures (Fig. 2). The strong correlation between sludge CAG content and microbial composition suggested distinctive carbohydrate pathways encoded by microbes of different taxa in sludge microbiome and the incidence of CAGs’ horizontal gene transfer (HGT) is not frequent enough to obscure their association with genomes. This notion of infrequent HGT of CAGs was also supported by the largely consistent (62.2 ± 5 %) phylogenetic affiliation of neighboring genes of CAG-ORFs.

### Network analysis

Correlation-based network, in the form of a set of nodes joined in pairs by edges, carries meaningful topological features that may shed light on the underlying determinative interactions driving the system functions [15]. Here, we applied the co-occurrence network analysis to further resolve the capacities of microorganisms involved in carbohydrate metabolism.

For the sludge microbial network, a total of 165 pairs of significant and robust correlations, 75 positive and 90 negative correlations, were identified among the 46 major CAG families and 40 major microbial orders (Additional file 1: Tables S5, S6). As shown in Additional file 1: Figure S7, the whole network has 68 nodes of 14 phyla and 153 edges with average degree (the number of connections it has to other nodes) of 4.85. Clustering coefficient (CC) and modularity (MD) observed in the whole network and positive network were all higher than that of the ErdöS–Réyni random network with identical size (Table 2), suggesting the firm modular topology of these networks [16]. In addition, the high CC/CC-random ratio of 9.4 of the positive network strongly supported the ‘small world’ characteristic in which most nodes could be reached from every other by a small number of hubs [17]. In contrast, the negative network, which reflects exclusion pattern among nodes, tends to be scattered (an average clustering coefficient of 0) and less modularized compared to positive network (Table 2), suggesting distinct characteristics of positive and negative interactions. Besides, the dominant microbial groups of *Actinomycetales*, *Clostridiales* and *Thermotogales* (respectively, showed degree of 14, 12 and 11 in the whole network), minor populations, such as *Sphingomonadales*, *Xanthomonadales*, *Rhizobiales* and methanogenic *Methanosarcinales* (respectively, with degree of 12, 16, 13 and 11 in the whole network), also served as the connection centers of the network, suggesting their keystone involvements in carbohydrate metabolism of sludge microbiota (Additional file 1: Figure S7).

**Table 2:** Topological properties of co-occurrence/co-exclusion networks of sludge microbiomes collected from WWTPs

	Modularity	Clustering coefficient CC	Average path length APL	Network diameter ND	Average degree AD	Graph density GD
Whole network (68 nodes, 165 edges)	0.48	0.49	2.89	7.5	4.85	0.07
Corresponding random network	0.39 ± 0.015	0.07 ± 0.016	2.79 ± 0.039	5.56 ± 0.57	4.85	0.07
Positive network (53 nodes, 75 edges)	0.66	0.47	2.53	5.88	2.83	0.05
Corresponding random network	0.52 ± 0.024	0.05 ± 0.027	3.63 ± 0.193	8.12 ± 1.029	2.83	0.05
Negative network (56 nodes, 90 edges)	0.50	0	2.87	6.7	3.21	0.06
Corresponding random network	0.49 ± 0.021	0.06 ± 0.023	3.39 ± 0.123	7.44 ± 0.848	3.21	0.06

**Fig. 3:**
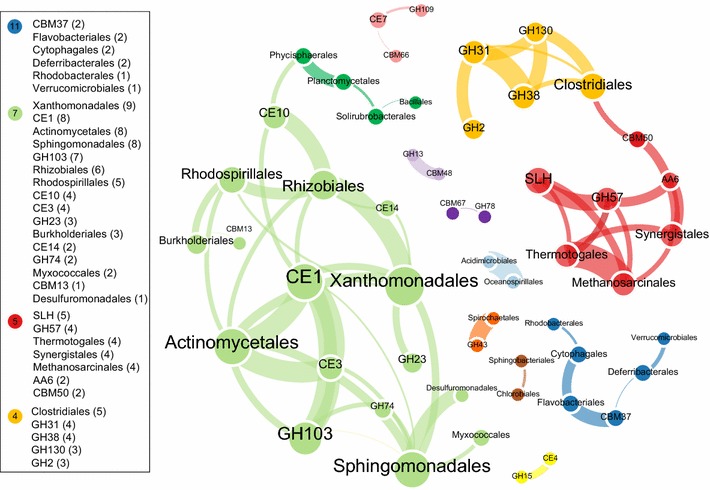
Co-occurrence network (that is the positive network) among 46 major CAG families and 40 prevalent phylogenetic orders. Nodes representing either CAG families or phylogenetic orders, were colored according to the network modules (that is clusters) determined by multi-level aggregation method (Louvain algorithm), while edge indicating a strong (Spearman’s rank correlation coefficient *r* ^2^ > 0.6) and significant (*p* value <0.01) positive correlation between node pairs, is in the same color with its source node. The size of each node and the font size of label is proportion to the number of connections (that is degree) of that node. And the *thickness* of edge is proportion to the correlation coefficient between nodes

Once the network assembly was demonstrated to be modular, further investigation was conducted to explore the co-occurrence/co-exclusion pattern in the positive and negative sub-networks. In all likelihood, positive correlations between CAG families could originated either from ecological symbioses between species possessing one of the correlated enzymes, or association of correlated enzymes in one single species, and the co-occurrence between CAG families and taxa may help to resolve these two possibilities. Further looking into the positive network modules determined by multi-level aggregation method [18], we observed that nodes in four modules showed co-occurring incidence higher than expected in random association (Additional file 1: Tables S7, S8). As illustrated in Fig. 3, the prevalent *Actinomycetales*, *Clostridiales* and *Thermotogales,* respectively, developed into hubs of three interrelated network modules coupled with distinguishable carbohydrate-related metabolic pathways. This interaction network of sludge microbiome is quite distinct from that of human and ruminal animal’s gut in which the dominance of *Firmicutes* and *Bacteroidetes* [1] varied due to the presence or absence of the methanogenic archaea [19]. Cluster *Clostridiales* inter-connected with GH130, GH38, GH31 and GH2 which catalyze the hydrolysis of both alpha- and beta-linked saccharides, suggest the wide substrate spectrum of *Clostridiales* as a primary digestive population in the sludge microbiome. The module containing thermostable GH57 family [20, 21] and populations of *Thermotogales,Methanosarcinales, Synergistales,* in which thermos-tolerant strains had been commonly reported [22–24], demonstrated a thermophilic ecological niche within the sludge community. The linkage of *Synergistales* with AA6 suggested its putative lingnolytic capacity to degrade lignin which is found invariably with polysaccharides in plant cell wall. Interestingly, strong association was observed between GH57 and S-layer homology domains (SLH) both of which interlinked with the *Thermotogales* population in the network. This association was in agreement with the phylogenetic annotation of ORFs encoding GH57 and SLH domains that we observed these two enzyme families coexisted in *Thermotogales* in five (out of nine) metagenomes studied. Since current available genomes of *Thermotogales* does not show such a co-occurrence pattern (according to association search in CAT database), there might present some novel *Thermotogales* population encompassing a GH57 and SLH associated pathway. However, we cannot obtain direct evidence for this GH57–SLH association in *Thermotogales*’ genome since no contig containing both of these two families could be identified in current assembly. Further retrieving genome bins of *Thermotogales* might help to resolve the possibility for the association. Noteworthy, the module containing *Actinomycetales*, *Xanthomonadales* and *Sphinogmonadales* were characterized by the involvement of a series of carbohydrate esterases (CEs) families which could catalyze the hydrolysis of acetyl groups from polymeric xylan. And the solid correlation among *Actinomycetales*, CE1, CE3 as well as the peptidoglycan lyase (GH103) indicated the putative function of *Actinomycetales* in debranching hemicellulose and peptidoglycan hydrolysis.

The appendance of CBM48 to GH13 was revealed as a robust positive correlation between these two families (Fig. 3), which was in agreement with the crystal structure of amylolytic enzymes of GH13 families [25, 26]. Furthermore, the finding of catalytic mechanisms of GH78 (SaRha78A) in *Streptomyces avermitilis* [27] confirmed the positive co-occurrence pattern observed between α-l-rhamnosidase (GH78) and CBM67, though the putative microbial hosts of these enzyme pairs could not be identified in the metagenomes studied. The agreement of enzymatic cooperation revealed by structural characterization [27] and by co-occurrence pattern observed in the network authenticated the assumption that enzymes steadily work together should co-occur more often than expected by chance in network. Therefore, the co-occurrence pattern may serve as an indicator for discovering synergistic enzyme association in uncultured microbes. However, there still present some intriguing enzymatic association that cannot be fully elucidated by data at hand, for example the co-occurrence of CBM37 with *Flavobacteriales.* This newly characterized CBM37 domain [28, 29], enabling cellulosome-independent attachment to cellulosic substrates, is by now identified exclusively in genus of *Ruminococcus* from *Clostridiales.* The association between CBM37 and *Flavobacteriales* led us to the speculation of some unnoticed possessing of CBM37 domain in the *Fibrobacter* genus which showed close ecological niche with *Ruminococcus* in ruminal microbiome. However, CBM37 domain was absent from the three available genomes of *Fibrobacter.* Although CBM37-encoding ORFs did not show homogenous phylogenetic affiliation to *Flavobacteriales* by the LCA algorithm applied, it is still plausible that some unknown species of *Flavobacteriales* also mediate cell surface attachment via CBM37-like domain; and further genome recovery is required to validate this speculation.

Comparing to the positive correlation, the co-exclusion pattern in the negative network could be useful to identify the possible microbial competitions among species (Additional file 1: Figure S8). The unclustered and less modularized structure of negative network suggested that negative interactions (for example, competition) among microorganisms were established by relatively open ‘one-to-many’ or ‘one-to-one’ exclusion. We observed in the negative network a mutual exclusive pattern between anaerobic and aerobic microbes, for example *Clostridiales* against *Actinomycetales,* mirroring their different niches of dissolved oxygen in WWTPs. In addition, exclusive pattern was not observed between anaerobic-prevalent *Thermotogales* and *Clostridiales*, suggesting their noncompetitive relationship within the anaerobic niche. Given the general heterotrophic life styles of these populations, the virtual independence of *Clostridiales*-leading and *Thermotogales*-leading module in both positive and negative network (Fig. 3, Additional file 1: Figure S8) revealed their distinct carbon metabolism probably on different substrates or with different mechanisms.

### Limitations

Although the metagenomic analyses have revealed some interesting patterns on community assembly and CAGs’ association, we would like to point out a few technical limitations which may affect these results. First, the short reads length (100 bp) may hinder precise classification of 16S rRNA genes though MEGAN indicates 100 bp is long enough to identify a species [30]. Moreover, overaggressive CAG prediction may be caused by the HMM-based annotation method even though a better sensitivity and accuracy than similarity-based (BLAST) annotation approach had been reported [31]. Finally, although NGS-based metagenomics has no PCR-related biases, it is still being limited by other factors, including DNA extraction method, sequencing depth and assembly strategy [30].

## Conclusions

Comparative metagenomics coupled with metabolic network reconstruction revealed the abundance distribution, phylogenetic diversity as well as metabolic cooperation of carbohydrate-active genes within microbiomes collected from different units of WWTPs. Besides, the influence of dissolved oxygen and salinity, community phylogeny fundamentally structured the overall pathway of carbohydrate metabolism of sludge system. The predominant *Actinomycetales*, *Clostridiales* and *Thermotogales,* respectively, serve as the keystone population of distinctive pathway modules within the sludge carbohydrate metabolic network. Mutual exclusive pattern between aerobic and anaerobic microbes in the co-exclusive network highlighted the importance of niche differentiation by dissolved oxygen in sludge systems. Meanwhile the major anaerobic hydrolyzers of *Clostridiales* showed noncompetitive relationship with *Thermotogales* which potentially encoded S-layer associated GH57 family. Together, the enzymatic associations and inter-species cooperation patterns revealed here would serve as timely supplementation to expand the presently constrained understanding of carbohydrate metabolism in sludge microbiota.

## Methods

### Sample collection

Activated sludge (AS) was collected from two wastewater treatment plants, namely the Shatin Wastewater Treatment Plant (ST, Hong Kong SAR, China) and Stanley Wastewater Treatment Plant (Stanley, Hong Kong, SAR, China). The ST Plant was chosen to represent the saline wastewater treatment (generally having salinity of 1 %) as sea water has been used for flashing in major area of the city, while Stanley plant treating was selected to represent the treatment process treating fresh (common) domestic wastewater. For fresh AS process, both the biofilm and suspended AS were collected. Biofilm was scraped from the carrier inside the tank where suspended AS was collected. For saline AS process, sludge was collected from both winter and summer time of Hong Kong (Additional file 1: Table S9). The AS collected showed average chemical oxygen demand (COD) of 622 mg/L with removal efficiency of 90 %.

Anaerobic digestion sludge (ADS) was collected from ST and Shek Wu Hui (SWH) WWTPs representing the saline and fresh anaerobic sludge digestion processes, respectively. The ADS collected had average TS of 2000 mg/L (equivalent to COD of 52 mg/L) with reduction rate of 30 %. In addition to the sludge from full-scale anaerobic digesters, two lab-scale semi-continuous batch digesters with temperature precisely controlled at mesophilic condition (35 °C, MAD) and thermophilic condition (55 °C, TAD) were selected to investigate the effect of temperature on carbohydrate metabolism [9]. AD sludge of Shatin WWTP was used to seed these reactors. Finally, sludge from a thermophilic cellulose fermenter (TCF) fed solely on microcrystalline cellulose at 55 °C was added to the sample list as a reference for cellulose metabolism [10] (detailed sample description is listed in Table 1 and Table S9). AD sludge from Shek Wu Hui WWTP served as the seed for the TCF reactor.

### Technical and biological replicates

As shown in Additional file 1: Figure S1, to access the reproducibility of technical replicates, sludge sample from SWH anaerobic digester was subject to replicate DNA extraction followed by independent metagenomic library construction and Illumina Hiseq 2000 paired-end sequencing. In the meantime, two sets of biological replicates were investigated with sludge samples collected at the same location but at two different time points (Additional file 1: Figure S1). These biological replicates include sludge from ST and SWH anaerobic digesters (Additional file 1: Table S9).

### DNA extraction and quality control (QC) of metagenomic sequences

Genomic DNA was extracted from 500 mg sample with FastDNA SPIN Kit for Soil (MP Biomedicals, LLC, Illkirch, France). The concentration and quality of the extracted DNA was determined (Nanodrop, ND-1000, USA) and summarized in Additional file 1: Table S5. DNA library of ~180 bp was prepared and sequenced by BGI (Shenzhen, China) using Illumina HiSeq 2000 technology generating 2 × 100 bp paired-end reads following the Illumina’s instruction. Please refer to our previous publications for the technical details [32].

The paired-end sequences was firstly quality-checked by removing any read containing ambiguous base of letter N and then trimming off the sequencing adaptors to get reads of 100 bp in length. Next, artificial duplicates, showing 100 % identity over the first 50 base pairs, were filtered out from the dataset [33]. All the metagenomic datasets were deposited to MG-RAST server for data sharing (See Additional file 1: Table S9 for accession number).

### Metagenomic assembly and carbohydrate-active gene (CAG) prediction

The post-QC reads were firstly assembled using MetaVelvet (version 1.1.01) [34, 35] with kmer length of 51. The assembled contigs longer than 300 bp [36] were subject to gene prediction using MetaGeneMark (version 2.8) [37] with default parameters (Additional file 1: Table S6). Next, amino acid sequences of the predicted open reading frames (ORFs) were screened against Hidden Markov Models (HMMs) collected at dbCan [31] using hmmscan [38] with *E* value cutoff of 1E−4 [2] for CAGs families classified by the CAZy (Carbohydrate Active enZyme) database [39]. CAZymes Analysis Toolikt (CAT) [40] was used to check direct associations between CAG families.

### Taxonomic and functional annotation

Community structure was inferred based on both 16S/18S rRNA genes and protein-coding genes. 16S/18S rRNA sequences were identified by BLASTN (2.6.26+) [41] against Silva SSU rRNA database with *E* value cutoff of 1E−20 [42] (Table S1). Meanwhile, the annotation of the protein-coding reads were carried out in two steps: (1) the predicted ORFs were subjected to Rapsearch search (version 2.0) [43] against NCBI *nr* database (downloaded at Feb. 2015) with *E* value cutoff of 1E−5, and 2) reads were mapped to the ORFs with bowtie [44] allowing two mismatches. The number of reads mapped to each ORF was counted by the SAMtools package [45] and used to quantify ORF’s abundance within metagenome [46]. MEGAN4 [30] was used to parse the tabular output of BlASTN and Rapsearch into various taxonomic and functional (SEED) levels. For comparison purposes, all distributions were normalized as a function of the number of annotated sequences/genes.

### Statistical analysis of CAG families

To ensure confidence with statistical significance, CAG families and phylogenetic orders were first checked to filter out those families existing in less than half of the sludge samples and having relative abundance less than 1 % across all of the samples, resulting in 46 major CAG families and 40 major microbial orders (16S rRNA gene based) whose abundances are listed in Additional file 1: Tables S5 and S6. Such preliminary filtering step removing those poorly represented families greatly facilitated the detection of the core variation and interactions among families.

One-way ANOVA was conducted to evaluate the influence of environmental factors of temperature, DO, and salinity on CAG abundance profiles using “anova()” function implemented in R. Principal coordinate analysis (PCoA) was performed based on Bray–Curtis distances of CAG profiles and bacterial community composition built from either 16S rRNA genes data or CAG-encoding sequences with “procomp()” function. Procrustes transformations (using “procrustes()” function in VEGAN package) was conducted with two PCoA plots as input; one representing phylogenetic composition and the other representing the carbohydrate pathways. The significance (*p* value <0.05 [47]) of any Procrustes transformation was determined by comparing the measure of fit, *M*^2^ (the sum of square distance between matched sample pairs), between matched sample PCoA plots to a distribution of *M*^2^ values empirically determined from 10,000 label permutations using “protest()” function [47]. In addition to *p* value, Procrustes superimposition plot was used to evaluate the congruence between ordinations.

### Network analysis

Next, correlation matrix was constructed by calculating all pairwise Spearman’s rank correlation between the major 46 CAG families and 40 phylogenetic orders for network analysis. A robust correlation was a strong correlation with the Spearman’s correlation coefficient (*r*^2^) higher than 0.6 and *p* value (after multiple testing correction [48]) lower than 0.05 [49]. The correlation network was visualized in Gephi [50]. Positive and negative networks were, respectively, subseted from the whole network based on the positive and negative values of correlation. For topology comparison, 10,000 Erdös–Réyni random networks with the same number of nodes and edges as in the real network were generated [51]. A set of topological properties was calculated in R with igraph package [52] (Table 2).

Observed co-occurring incidence within a module was measured as relative percentage of observed edges within the module in the total 156 edges of the whole network, while the random co-occurring incidence was the theoretical incidence of co-occurrence calculated by considering the module frequencies and random association. The degree of disagreement between the observed and random co-occurring incidence was used as a benchmark for exploring the nonrandom assembly patterns within network [53].

## Additional file

10.1186/s13068-015-0348-2 16S/18S rRNA annotation efficiency of sludge samples collected from different process of WWTP. **Table S2.** Phylogenetic and functional correlation between technical and biological replicates (Pearson’s correlation coefficient). **Table S3.** All CAG families detected in the sludge samples. CAGs are quantified based on the number of ORFs containing the particular CAG domain. CAG families are sorted alphabetically according to their names. **Table S4.** Comparison of CAGs involves in lignocellulose hydrolysis between sludge system and other four plant feeding microbiota. Glycoside hydrolase (GH) families are assigned to enzyme categories based on the classification previously published [3]. **Table S5.** Quantification of 46 major CAG families detected based on ORFs containing the particular CAG domain. CAG families are sorted descending according to their average relative abundance across sludge samples. **Table S6.** Quantification of 40 major orders within the compared sludge samples based on 16S rRNA gene sequences. Orders are sorted descending according to their average relative abundance across sludge samples. **Table S7.** Topological properties of the co-occurrence network (positive network) of 46 major CAG families and 40 prevalent phylogenetic orders. Table S8. Observed and random co-occurring incidence within network modules. **Table S9.** Information of the metagenomic libraries of sludge samples and technical/biological replicates. **Table S10.** Statistics of assembled scaffolds from metagenome of sludge samples and technical/biological replicates. **Figure S1.** Illustration of the experimental design of metagenomes used for this study. Frames of technical and biological replicates are respectively filled with blue and green color. **Figure S2.** Rarefaction analysis of the sludge metagenomes. **Figure S3.** Phylogenetic orders showed significant variation (P-value< 0.05 and proportion difference > 1%) between biological replicates. **Figure S4.** Similarity distribution of GH-encoding ORFs to their best BLASTN hit against NCBI nr database. Left and right figure respectively shows the ORFs counts and accumulative abundance of GH-encoding ORFs from different sludge microbiomes. **Figure S5.** Heatmap of the most prevalent phylogenetic groups (order level) determined by the CAGs-encoding genes (a) and 16S rRNA gene sequences (b). **Figure S6.** Major CAG families showed significant variation (p-value < 0.05 by one-way ANOVA analysis) among sludge samples with different dissolved oxygen (left), temperature (middle) and salinity (right). Abbreviations in the figures: M: Mesophilic; A: Ambient temperature; T: Thermophilic. **Figure S7.** Whole network among 46 major CAG families and 40 prevalent phylogenetic orders. Nodes representing either CAG families or phylogenetic orders, are colored according to the network modules (that is clusters) determined by multi-level aggregation method (Louvain algorithm [18]). Each edge represents a strong (Spearman’s rank correlation coefficient r2 > 0.6) and significant (p-value < 0.01) correlation between node-pairs. Edges are colored according to the value of r2 with red stands for positive correlation; blue represents negative correlation. The size of each node and the font size of label is proportion to the number of connections (that is degree) of that node. And the thickness of edge is proportion to the correlation coefficient between nodes. **Figure S8.** Co-exclusion network (that is the negative network) among 46 major CAG families and 40 prevalent phylogenetic orders. Nodes representing either CAG families or phylogenetic orders, are colored according to the network modules (that is clusters) determined by multi-level aggregation method (Louvain algorithm [18]). Each edge representing a strong (Spearman’s rank correlation coefficient r2 > 0.6) and significant (p-value < 0.01) correlation between node-pairs, are in the same color with its source node. The size of each node and the font size of label is proportion to the number of connections (that is degree) of that node. And the thickness of edge is proportion to the correlation coefficient between nodes.

## Abbreviations

CAG: carbohydrate-active gene
WWTP: wastewater treatment plant
NGS: next-generation sequencing
AS: activated sludge
ADS: anaerobic digestion sludge
DO: dissolved oxygen
GH: glycoside hydrolase
CE: carbohydrate esterase
CBM: carbohydrate-binding module
HGT: horizontal gene transfer
CC: clustering coefficient
MD: modularity
