# Supplementary material for: Phylogeny-structured carbohydrate metabolism across microbiomes collected from different units in wastewater treatment process
Source: Biotechnol Biofuels. 2015 Oct 22;8:172. doi: 10.1186/s13068-015-0348-2 (PMC4618737; doi:10.1186/s13068-015-0348-2)
Supplement: Supplementary file 1 — 10.1186/s13068-015-0348-2 16S/18S rRNA annotation efficiency of sludge samples collected from different process of WWTP. Table S2. Phylogenetic and functional correlation between technical and biological replicates (Pearson’s correlation coefficient). Table S3. All CAG families detected in the sludge samples. CAGs are quantified based on the number of ORFs containing the particular CAG domain. CAG families are sorted alphabetically according to their names. Table S4. Comparison of CAGs involves in lignocellulose hydrolysis between sludge system and other four plant feeding microbiota. Glycoside hydrolase (GH) families are assigned to enzyme categories based on the classification previously published [3]. Table S5. Quantification of 46 major CAG families detected based on ORFs containing the particular CAG domain. CAG families are sorted descending according to their average relative abundance across sludge samples. Table S6. Quantification of 40 major orders within the compared sludge samples based on 16S rRNA gene sequences. Orders are sorted descending according to their average relative abundance across sludge samples. Table S7. Topological properties of the co-occurrence network (positive network) of 46 major CAG families and 40 prevalent phylogenetic orders. Table S8. Observed and random co-occurring incidence within network modules. Table S9. Information of the metagenomic libraries of sludge samples and technical/biological replicates. Table S10. Statistics of assembled scaffolds from metagenome of sludge samples and technical/biological replicates. Figure S1. Illustration of the experimental design of metagenomes used for this study. Frames of technical and biological replicates are respectively filled with blue and green color. Figure S2. Rarefaction analysis of the sludge metagenomes. Figure S3. Phylogenetic orders showed significant variation (P-value< 0.05 and proportion difference > 1%) between biological replicates. Figure S4. Similarity distribution o [file 13068_2015_348_MOESM1_ESM.docx]

1. 16S/18S rRNA annotation efficiency of sludge samples collected from different process of WWTP.

| **Sample name** | **rRNA percentage** | **Domain** | **Phylum** | **Class** | **Order** | **Family** | **Genus** |
| --- | --- | --- | --- | --- | --- | --- | --- |
| **Stanley_AS** | 0.06% | 99.99% | 72.79% | 63.42% | 51.72% | 32.50% | 20.99% |
| **Stanley_BF** | **0.01%** | 99.98% | 68.12% | 61.37% | 46.18% | 31.16% | 18.93% |
| **ST_AS_summer** | 0.06% | 96.68% | 48.94% | 37.29% | 24.83% | 13.88% | 5.78% |
| **ST_AS_winter** | 0.06% | 97.45% | 62.59% | 51.17% | 35.93% | 21.41% | 8.52% |
| **ST_ADS** | 0.07% | 98.70% | 49.72% | 37.84% | 30.24% | 21.99% | 11.23% |
| **SWH_ADS** | 0.08% | 98.66% | 49.45% | 41.80% | 33.04% | 23.80% | 12.69% |
| **MAD** | 0.08% | 98.91% | 50.65% | 37.46% | 30.06% | 19.52% | 8.99% |
| **TAD** | 0.08% | 98.96% | 45.82% | 39.64% | 25.37% | 16.08% | 7.59% |
| **TCF** | 0.09% | 99.20% | 67.29% | 62.31% | 46.27% | 39.06% | 23.39% |

1. Phylogenetic and functional correlation between technical and biological replicates (Pearson’s correlation coefficient).

| Replicate 1 | Replicate 2 | Order^1)^ | SEED Level2 |
| --- | --- | --- | --- |
| ADS_SWH_2012-3_1 | SWH_ADS | 1.00 | 1.00 |
| ADS_ST_2011-9 | ST_ADS | 0.85 | 0.97 |
| ADS_SWH_2011-9 | SWH_ADS | 0.80 | 0.99 |

1. Annotated based on homology search of reads by NCBI BLASTN against SSUref115 database with e-value cut-off of 1E-20
2. All CAG families detected in the sludge samples. CAGs are quantified based on the number of ORFs containing the particular CAG domain. CAG families are sorted alphabetically according to their names.

| **CAG family** | **Stanley_AS** | **Stanley_BF** | **ST_AS**  **summer** | **ST_AS**  **winter** | **ST_ADS** | **SWH_ADS** | **MAD** | **TAD** | **TCF** |
| --- | --- | --- | --- | --- | --- | --- | --- | --- | --- |
| **AA1** | 4 | 3 | 1 | 0 | 0 | 0 | 1 | 0 | 0 |
| **AA10** | 2 | 3 | 1 | 0 | 0 | 0 | 0 | 0 | 0 |
| **AA2** | 106 | 112 | 29 | 31 | 17 | 10 | 7 | 10 | 5 |
| **AA3** | 117 | 130 | 12 | 8 | 2 | 6 | 3 | 5 | 6 |
| **AA4** | 55 | 81 | 7 | 5 | 6 | 10 | 6 | 7 | 9 |
| **AA5** | 5 | 2 | 0 | 0 | 0 | 0 | 0 | 0 | 0 |
| **AA6** | 133 | 157 | 34 | 28 | 73 | 51 | 28 | 31 | 70 |
| **AA7** | 104 | 142 | 11 | 17 | 4 | 5 | 7 | 6 | 13 |
| **AA9** | 0 | 0 | 0 | 1 | 0 | 0 | 0 | 0 | 0 |
| **CBM1** | 0 | 1 | 0 | 0 | 0 | 0 | 0 | 0 | 0 |
| **CBM10** | 2 | 1 | 0 | 0 | 0 | 0 | 0 | 0 | 0 |
| **CBM11** | 8 | 2 | 4 | 10 | 2 | 2 | 2 | 0 | 0 |
| **CBM12** | 22 | 10 | 4 | 4 | 10 | 5 | 5 | 2 | 1 |
| **CBM13** | 97 | 42 | 13 | 15 | 4 | 98 | 2 | 0 | 7 |
| **CBM14** | 8 | 8 | 0 | 3 | 0 | 2 | 0 | 1 | 0 |
| **CBM15** | 4 | 2 | 1 | 2 | 2 | 0 | 0 | 0 | 0 |
| **CBM16** | 85 | 74 | 24 | 22 | 10 | 27 | 11 | 13 | 17 |
| **CBM17** | 0 | 0 | 0 | 0 | 0 | 1 | 0 | 1 | 0 |
| **CBM18** | 2 | 1 | 0 | 0 | 0 | 0 | 0 | 0 | 0 |
| **CBM19** | 1 | 0 | 0 | 0 | 0 | 0 | 0 | 0 | 0 |
| **CBM2** | 52 | 32 | 16 | 11 | 6 | 6 | 0 | 1 | 6 |
| **CBM20** | 71 | 30 | 32 | 32 | 10 | 19 | 8 | 6 | 20 |
| **CBM21** | 8 | 1 | 3 | 0 | 0 | 1 | 0 | 0 | 0 |
| **CBM22** | 37 | 27 | 9 | 8 | 4 | 13 | 8 | 7 | 13 |
| **CBM23** | 10 | 5 | 5 | 1 | 3 | 3 | 4 | 1 | 0 |
| **CBM24** | 1 | 1 | 0 | 0 | 0 | 0 | 0 | 0 | 0 |
| **CBM25** | 2 | 2 | 3 | 0 | 0 | 1 | 4 | 1 | 4 |
| **CBM26** | 9 | 2 | 2 | 3 | 1 | 2 | 1 | 0 | 0 |
| **CBM27** | 0 | 0 | 0 | 0 | 1 | 1 | 0 | 0 | 0 |
| **CBM28** | 0 | 0 | 1 | 1 | 0 | 0 | 0 | 1 | 0 |
| **CBM29** | 1 | 1 | 0 | 1 | 0 | 0 | 0 | 0 | 0 |
| **CBM3** | 1 | 0 | 2 | 0 | 0 | 2 | 0 | 0 | 20 |
| **CBM30** | 15 | 2 | 4 | 3 | 5 | 5 | 5 | 1 | 2 |
| **CBM31** | 0 | 1 | 0 | 1 | 0 | 1 | 0 | 0 | 1 |
| **CBM32** | 311 | 155 | 72 | 71 | 49 | 60 | 32 | 25 | 35 |
| **CBM34** | 19 | 9 | 3 | 2 | 2 | 7 | 12 | 5 | 4 |
| **CBM35** | 145 | 92 | 30 | 23 | 8 | 17 | 14 | 12 | 20 |
| **CBM36** | 5 | 5 | 2 | 0 | 1 | 2 | 1 | 0 | 1 |
| **CBM37** | 97 | 33 | 43 | 34 | 16 | 18 | 14 | 7 | 16 |
| **CBM38** | 30 | 21 | 5 | 3 | 0 | 3 | 6 | 5 | 1 |
| **CBM39** | 2 | 0 | 1 | 1 | 0 | 1 | 0 | 1 | 1 |
| **CBM4** | 31 | 13 | 10 | 10 | 8 | 11 | 3 | 4 | 3 |
| **CBM40** | 171 | 88 | 37 | 32 | 14 | 19 | 16 | 26 | 35 |
| **CBM41** | 13 | 10 | 0 | 7 | 2 | 2 | 2 | 6 | 3 |
| **CBM42** | 9 | 2 | 1 | 0 | 1 | 1 | 0 | 0 | 4 |
| **CBM44** | 798 | 277 | 317 | 199 | 191 | 262 | 78 | 58 | 179 |
| **CBM45** | 1 | 0 | 1 | 1 | 1 | 0 | 0 | 0 | 3 |
| **CBM46** | 6 | 5 | 3 | 0 | 2 | 3 | 0 | 1 | 0 |
| **CBM47** | 38 | 27 | 6 | 1 | 0 | 0 | 0 | 10 | 1 |
| **CBM48** | 367 | 280 | 78 | 98 | 43 | 61 | 47 | 35 | 30 |
| **CBM49** | 1 | 0 | 1 | 1 | 1 | 1 | 0 | 0 | 0 |
| **CBM5** | 8 | 19 | 4 | 2 | 3 | 9 | 1 | 0 | 0 |
| **CBM50** | 615 | 633 | 240 | 239 | 135 | 193 | 120 | 154 | 232 |
| **CBM51** | 29 | 33 | 1 | 5 | 1 | 8 | 3 | 8 | 17 |
| **CBM52** | 0 | 0 | 0 | 2 | 0 | 0 | 0 | 0 | 0 |
| **CBM53** | 2 | 1 | 3 | 3 | 0 | 1 | 1 | 0 | 0 |
| **CBM54** | 7 | 5 | 4 | 0 | 4 | 8 | 0 | 4 | 4 |
| **CBM55** | 2 | 1 | 0 | 0 | 1 | 1 | 0 | 0 | 0 |
| **CBM56** | 38 | 11 | 7 | 3 | 4 | 7 | 50 | 1 | 6 |
| **CBM57** | 43 | 42 | 7 | 7 | 5 | 2 | 1 | 6 | 3 |
| **CBM58** | 2 | 0 | 0 | 0 | 2 | 0 | 0 | 0 | 0 |
| **CBM59** | 2 | 1 | 2 | 0 | 0 | 0 | 0 | 1 | 0 |
| **CBM6** | 63 | 33 | 21 | 18 | 8 | 11 | 5 | 6 | 27 |
| **CBM60** | 6 | 2 | 0 | 1 | 0 | 1 | 1 | 3 | 0 |
| **CBM61** | 32 | 13 | 10 | 9 | 4 | 11 | 4 | 8 | 5 |
| **CBM62** | 1 | 0 | 0 | 0 | 0 | 1 | 1 | 0 | 1 |
| **CBM63** | 15 | 6 | 3 | 8 | 3 | 2 | 2 | 0 | 2 |
| **CBM64** | 0 | 1 | 1 | 0 | 1 | 0 | 0 | 0 | 0 |
| **CBM65** | 3 | 3 | 1 | 1 | 1 | 0 | 0 | 0 | 0 |
| **CBM66** | 232 | 204 | 42 | 45 | 20 | 25 | 27 | 43 | 39 |
| **CBM67** | 157 | 82 | 28 | 24 | 6 | 28 | 3 | 30 | 26 |
| **CBM8** | 15 | 16 | 4 | 2 | 2 | 0 | 1 | 0 | 1 |
| **CBM9** | 160 | 143 | 54 | 49 | 21 | 32 | 15 | 12 | 51 |
| **CE1** | 1449 | 1314 | 300 | 273 | 120 | 162 | 99 | 85 | 115 |
| **CE10** | 824 | 945 | 147 | 131 | 68 | 101 | 58 | 66 | 60 |
| **CE11** | 112 | 115 | 33 | 34 | 20 | 10 | 11 | 9 | 15 |
| **CE12** | 78 | 41 | 26 | 20 | 4 | 9 | 9 | 9 | 20 |
| **CE13** | 20 | 16 | 2 | 1 | 1 | 1 | 0 | 0 | 1 |
| **CE14** | 285 | 271 | 77 | 71 | 26 | 35 | 33 | 32 | 44 |
| **CE15** | 82 | 48 | 14 | 8 | 3 | 9 | 5 | 15 | 9 |
| **CE16** | 20 | 22 | 2 | 2 | 1 | 1 | 0 | 1 | 1 |
| **CE2** | 28 | 24 | 6 | 4 | 5 | 3 | 2 | 2 | 2 |
| **CE3** | 253 | 231 | 52 | 45 | 22 | 35 | 19 | 10 | 25 |
| **CE4** | 562 | 665 | 108 | 87 | 63 | 101 | 44 | 61 | 66 |
| **CE5** | 67 | 96 | 8 | 22 | 0 | 18 | 9 | 8 | 0 |
| **CE6** | 106 | 42 | 17 | 13 | 9 | 7 | 13 | 13 | 9 |
| **CE7** | 139 | 131 | 13 | 25 | 11 | 11 | 11 | 20 | 28 |
| **CE8** | 42 | 24 | 15 | 14 | 10 | 14 | 11 | 3 | 11 |
| **CE9** | 69 | 55 | 7 | 3 | 7 | 23 | 12 | 13 | 12 |
| **Cohesin** | 64 | 42 | 22 | 10 | 12 | 37 | 5 | 7 | 36 |
| **Dockerin** | 121 | 85 | 15 | 29 | 34 | 41 | 5 | 37 | 57 |
| **GH1** | 105 | 93 | 22 | 22 | 7 | 25 | 8 | 17 | 25 |
| **GH10** | 57 | 31 | 8 | 7 | 5 | 14 | 4 | 4 | 18 |
| **GH100** | 5 | 2 | 2 | 2 | 0 | 0 | 1 | 1 | 4 |
| **GH102** | 59 | 72 | 5 | 5 | 2 | 1 | 1 | 1 | 4 |
| **GH103** | 106 | 173 | 20 | 24 | 9 | 4 | 5 | 1 | 4 |
| **GH104** | 10 | 10 | 2 | 0 | 3 | 4 | 2 | 1 | 0 |
| **GH105** | 30 | 12 | 14 | 10 | 6 | 11 | 5 | 1 | 11 |
| **GH106** | 12 | 4 | 3 | 1 | 2 | 9 | 1 | 4 | 15 |
| **GH108** | 38 | 33 | 6 | 4 | 3 | 10 | 6 | 1 | 6 |
| **GH109** | 1122 | 852 | 173 | 158 | 72 | 159 | 79 | 165 | 184 |
| **GH11** | 7 | 0 | 0 | 1 | 0 | 1 | 0 | 0 | 10 |
| **GH110** | 1 | 0 | 0 | 0 | 0 | 0 | 0 | 1 | 0 |
| **GH112** | 0 | 0 | 0 | 0 | 0 | 0 | 2 | 0 | 0 |
| **GH113** | 19 | 21 | 15 | 10 | 0 | 4 | 2 | 3 | 14 |
| **GH114** | 31 | 65 | 12 | 11 | 4 | 9 | 3 | 2 | 4 |
| **GH115** | 6 | 0 | 0 | 0 | 4 | 1 | 3 | 0 | 3 |
| **GH116** | 9 | 7 | 2 | 1 | 1 | 4 | 3 | 1 | 4 |
| **GH117** | 15 | 7 | 5 | 4 | 0 | 0 | 2 | 1 | 1 |
| **GH119** | 0 | 0 | 0 | 0 | 0 | 0 | 1 | 0 | 0 |
| **GH12** | 1 | 2 | 2 | 4 | 1 | 1 | 1 | 0 | 0 |
| **GH120** | 20 | 13 | 4 | 9 | 11 | 6 | 4 | 3 | 3 |
| **GH121** | 0 | 0 | 0 | 0 | 0 | 0 | 0 | 1 | 2 |
| **GH122** | 0 | 1 | 0 | 0 | 0 | 0 | 0 | 0 | 0 |
| **GH123** | 2 | 0 | 0 | 0 | 0 | 1 | 2 | 1 | 6 |
| **GH124** | 0 | 0 | 0 | 0 | 0 | 1 | 0 | 0 | 11 |
| **GH125** | 14 | 5 | 0 | 0 | 2 | 1 | 3 | 2 | 5 |
| **GH127** | 42 | 8 | 10 | 4 | 9 | 17 | 11 | 10 | 25 |
| **GH128** | 9 | 10 | 8 | 5 | 1 | 1 | 2 | 2 | 3 |
| **GH129** | 0 | 0 | 0 | 0 | 0 | 1 | 0 | 1 | 4 |
| **GH13** | 695 | 592 | 173 | 211 | 102 | 128 | 105 | 86 | 106 |
| **GH130** | 49 | 34 | 13 | 14 | 10 | 19 | 26 | 11 | 39 |
| **GH15** | 114 | 174 | 15 | 15 | 12 | 17 | 8 | 9 | 12 |
| **GH16** | 126 | 65 | 34 | 38 | 11 | 32 | 27 | 6 | 21 |
| **GH17** | 8 | 24 | 4 | 1 | 0 | 0 | 1 | 0 | 2 |
| **GH18** | 69 | 41 | 15 | 13 | 7 | 22 | 14 | 18 | 20 |
| **GH19** | 36 | 15 | 2 | 4 | 1 | 10 | 0 | 3 | 0 |
| **GH2** | 56 | 22 | 22 | 6 | 5 | 28 | 11 | 12 | 57 |
| **GH20** | 59 | 25 | 19 | 13 | 13 | 25 | 16 | 4 | 19 |
| **GH22** | 5 | 2 | 2 | 0 | 0 | 0 | 0 | 0 | 0 |
| **GH23** | 552 | 694 | 153 | 131 | 68 | 85 | 65 | 41 | 68 |
| **GH24** | 67 | 54 | 7 | 5 | 5 | 14 | 8 | 2 | 3 |
| **GH25** | 83 | 71 | 16 | 15 | 13 | 20 | 8 | 7 | 5 |
| **GH26** | 28 | 42 | 3 | 6 | 5 | 4 | 5 | 4 | 8 |
| **GH27** | 16 | 2 | 5 | 2 | 2 | 3 | 2 | 0 | 8 |
| **GH28** | 50 | 18 | 15 | 9 | 5 | 17 | 9 | 15 | 14 |
| **GH29** | 133 | 40 | 38 | 27 | 13 | 18 | 15 | 12 | 25 |
| **GH3** | 328 | 273 | 96 | 74 | 57 | 86 | 50 | 33 | 69 |
| **GH30** | 37 | 11 | 16 | 6 | 9 | 18 | 8 | 2 | 13 |
| **GH31** | 90 | 64 | 23 | 17 | 12 | 23 | 14 | 14 | 32 |
| **GH32** | 98 | 37 | 13 | 19 | 0 | 16 | 6 | 9 | 8 |
| **GH33** | 160 | 118 | 46 | 55 | 4 | 15 | 12 | 27 | 27 |
| **GH35** | 22 | 11 | 2 | 2 | 11 | 8 | 7 | 0 | 11 |
| **GH36** | 20 | 16 | 2 | 3 | 1 | 8 | 6 | 2 | 16 |
| **GH37** | 14 | 6 | 4 | 3 | 0 | 2 | 1 | 3 | 1 |
| **GH38** | 44 | 49 | 17 | 12 | 9 | 17 | 16 | 13 | 32 |
| **GH39** | 67 | 68 | 16 | 30 | 0 | 14 | 16 | 16 | 27 |
| **GH4** | 68 | 62 | 14 | 20 | 4 | 14 | 15 | 24 | 36 |
| **GH42** | 38 | 37 | 5 | 4 | 1 | 12 | 3 | 6 | 18 |
| **GH43** | 160 | 45 | 46 | 35 | 27 | 41 | 22 | 14 | 58 |
| **GH44** | 21 | 13 | 2 | 3 | 1 | 0 | 1 | 1 | 2 |
| **GH45** | 1 | 0 | 0 | 0 | 0 | 0 | 0 | 0 | 0 |
| **GH46** | 8 | 4 | 1 | 0 | 0 | 1 | 0 | 0 | 1 |
| **GH47** | 4 | 1 | 4 | 3 | 0 | 0 | 0 | 0 | 0 |
| **GH48** | 0 | 0 | 0 | 0 | 0 | 0 | 0 | 0 | 2 |
| **GH5** | 196 | 119 | 29 | 28 | 15 | 28 | 18 | 18 | 47 |
| **GH50** | 4 | 1 | 0 | 0 | 0 | 0 | 0 | 1 | 3 |
| **GH51** | 35 | 6 | 12 | 6 | 4 | 7 | 5 | 6 | 27 |
| **GH52** | 0 | 0 | 0 | 0 | 0 | 0 | 0 | 0 | 2 |
| **GH53** | 26 | 1 | 11 | 5 | 3 | 0 | 3 | 1 | 7 |
| **GH54** | 3 | 1 | 0 | 0 | 0 | 0 | 1 | 0 | 0 |
| **GH55** | 4 | 1 | 0 | 0 | 0 | 0 | 0 | 0 | 0 |
| **GH56** | 4 | 3 | 0 | 0 | 0 | 0 | 0 | 0 | 0 |
| **GH57** | 60 | 72 | 14 | 11 | 20 | 41 | 11 | 12 | 34 |
| **GH59** | 0 | 1 | 0 | 0 | 0 | 0 | 1 | 0 | 0 |
| **GH6** | 10 | 5 | 0 | 6 | 0 | 2 | 1 | 0 | 0 |
| **GH62** | 5 | 0 | 0 | 0 | 0 | 0 | 0 | 1 | 0 |
| **GH63** | 13 | 15 | 2 | 2 | 1 | 0 | 1 | 1 | 6 |
| **GH64** | 2 | 1 | 1 | 0 | 0 | 1 | 0 | 3 | 1 |
| **GH65** | 25 | 19 | 15 | 13 | 5 | 11 | 12 | 7 | 13 |
| **GH66** | 10 | 7 | 0 | 0 | 0 | 1 | 0 | 1 | 2 |
| **GH67** | 10 | 0 | 2 | 1 | 2 | 3 | 2 | 2 | 5 |
| **GH68** | 1 | 2 | 0 | 0 | 0 | 0 | 0 | 0 | 0 |
| **GH71** | 4 | 2 | 0 | 0 | 0 | 0 | 0 | 0 | 0 |
| **GH72** | 0 | 0 | 0 | 0 | 1 | 0 | 0 | 0 | 0 |
| **GH73** | 78 | 76 | 31 | 15 | 22 | 10 | 17 | 6 | 6 |
| **GH74** | 912 | 512 | 419 | 314 | 72 | 71 | 53 | 22 | 95 |
| **GH75** | 7 | 5 | 0 | 2 | 0 | 0 | 0 | 0 | 0 |
| **GH76** | 16 | 22 | 5 | 4 | 1 | 2 | 2 | 3 | 3 |
| **GH77** | 74 | 75 | 11 | 19 | 7 | 17 | 13 | 11 | 18 |
| **GH78** | 67 | 48 | 16 | 9 | 7 | 34 | 4 | 14 | 38 |
| **GH79** | 5 | 5 | 1 | 1 | 0 | 1 | 0 | 0 | 0 |
| **GH8** | 30 | 28 | 1 | 4 | 2 | 1 | 0 | 1 | 7 |
| **GH80** | 5 | 3 | 3 | 0 | 1 | 0 | 1 | 0 | 0 |
| **GH81** | 2 | 3 | 1 | 0 | 0 | 0 | 0 | 3 | 1 |
| **GH82** | 0 | 1 | 1 | 0 | 0 | 0 | 0 | 0 | 0 |
| **GH84** | 13 | 15 | 0 | 0 | 1 | 2 | 2 | 2 | 2 |
| **GH85** | 0 | 0 | 0 | 0 | 0 | 3 | 0 | 0 | 1 |
| **GH87** | 1 | 2 | 1 | 0 | 0 | 0 | 0 | 0 | 1 |
| **GH88** | 25 | 12 | 5 | 4 | 0 | 4 | 0 | 1 | 4 |
| **GH89** | 0 | 1 | 0 | 0 | 0 | 0 | 0 | 0 | 0 |
| **GH9** | 25 | 10 | 17 | 7 | 7 | 7 | 10 | 0 | 15 |
| **GH91** | 3 | 2 | 0 | 0 | 4 | 1 | 1 | 1 | 0 |
| **GH92** | 32 | 15 | 16 | 14 | 9 | 17 | 7 | 2 | 4 |
| **GH93** | 20 | 29 | 7 | 9 | 0 | 2 | 1 | 2 | 2 |
| **GH94** | 10 | 7 | 1 | 1 | 3 | 3 | 1 | 4 | 18 |
| **GH95** | 30 | 3 | 6 | 4 | 3 | 4 | 5 | 5 | 14 |
| **GH96** | 0 | 0 | 0 | 0 | 0 | 0 | 1 | 0 | 0 |
| **GH97** | 27 | 6 | 12 | 10 | 3 | 8 | 5 | 1 | 2 |
| **GH99** | 14 | 16 | 0 | 2 | 0 | 2 | 1 | 6 | 9 |
| **PL1** | 58 | 32 | 24 | 11 | 4 | 4 | 4 | 7 | 10 |
| **PL10** | 26 | 12 | 5 | 2 | 0 | 0 | 0 | 2 | 1 |
| **PL11** | 7 | 2 | 1 | 1 | 0 | 0 | 0 | 2 | 4 |
| **PL12** | 62 | 72 | 23 | 12 | 2 | 7 | 5 | 9 | 16 |
| **PL14** | 20 | 25 | 4 | 1 | 0 | 2 | 1 | 1 | 0 |
| **PL15** | 5 | 2 | 2 | 1 | 1 | 1 | 0 | 0 | 4 |
| **PL17** | 5 | 1 | 2 | 0 | 0 | 1 | 0 | 0 | 7 |
| **PL18** | 1 | 0 | 0 | 0 | 0 | 0 | 0 | 0 | 0 |
| **PL20** | 5 | 5 | 2 | 0 | 0 | 0 | 0 | 1 | 0 |
| **PL21** | 4 | 1 | 1 | 1 | 0 | 0 | 0 | 0 | 0 |
| **PL22** | 167 | 103 | 24 | 30 | 30 | 14 | 10 | 6 | 28 |
| **PL3** | 1 | 1 | 0 | 1 | 0 | 0 | 0 | 0 | 0 |
| **PL4** | 1 | 0 | 1 | 0 | 0 | 0 | 0 | 0 | 0 |
| **PL5** | 6 | 5 | 0 | 3 | 0 | 2 | 0 | 0 | 0 |
| **PL6** | 20 | 5 | 3 | 8 | 2 | 7 | 0 | 1 | 3 |
| **PL7** | 1 | 0 | 0 | 0 | 0 | 0 | 0 | 0 | 0 |
| **PL8** | 2 | 1 | 1 | 2 | 0 | 1 | 0 | 1 | 0 |
| **PL9** | 67 | 55 | 20 | 16 | 33 | 11 | 19 | 10 | 24 |
| **SLH** | 11 | 6 | 1 | 1 | 8 | 134 | 13 | 47 | 94 |

1. Comparison of CAGs involves in lignocellulose hydrolysis between sludge system and other four plant feeding microbiota. Glycoside hydrolase (GH) families are assigned to enzyme categories based on the classification previously published (Pope et al., 2010).

| CAZy families | Termite hindgut (Warnecke et al 2007) | Tammar wallaby (Pope et al 2010) | Rumen (Brulc et al 2009) | Rumen (Hess et al 2011) | Stanley_AS | Stanley_BF | AS_ST summer | | AS_ST winter | | ST_ ADS | | SWH_ADS | MAD | | TAD | | TCF | | |
| --- | --- | --- | --- | --- | --- | --- | --- | --- | --- | --- | --- | --- | --- | --- | --- | --- | --- | --- | --- | --- |
| **Endoglucannases** | | | | | | | | | | | | | | | | | | | |
| GH5 | 56 | 10 | 27 | 1451 | 196 | 119 | | 29 | | 28 | | 15 | 28 | 18 | 18 | | 47 | |
| GH6 | 0 | 0 | 0 | 0 | 10 | 5 | | 0 | | 6 | | 0 | 2 | 1 | 0 | | 0 | |
| GH7 | 0 | 0 | 0 | 1 | 0 | 0 | | 0 | | 0 | | 0 | 0 | 0 | 0 | | 0 | |
| GH9 | 9 | 0 | 24 | 795 | 25 | 10 | | 17 | | 7 | | 7 | 7 | 10 | 0 | | 15 | |
| GH45 | 4 | 0 | 0 | 115 | 1 | 0 | | 0 | | 0 | | 0 | 0 | 0 | 0 | | 0 | |
| GH48 | 0 | 0 | 1 | 3 | 0 | 0 | | 0 | | 0 | | 0 | 0 | 0 | 0 | | 2 | |
| Total | 69 | 10 | 52 | 2365 | 232 | 134 | | 46 | | 41 | | 22 | 38 | 29 | 18 | | 75 | |
| **Endohemicellulases** | | | | | | | | | | | | | | | | | | | |
| GH8 | 5 | 1 | 15 | 329 | 30 | 28 | | 1 | | 4 | | 2 | 1 | 0 | 1 | | 7 | |
| GH10 | 46 | 11 | 26 | 1025 | 57 | 31 | | 8 | | 7 | | 5 | 14 | 4 | 4 | | 18 | |
| GH11 | 14 | 0 | 3 | 165 | 7 | 0 | | 0 | | 1 | | 0 | 1 | 0 | 0 | | 10 | |
| GH12 | 0 | 0 | 0 | 0 | 1 | 2 | | 2 | | 4 | | 1 | 1 | 1 | 0 | | 0 | |
| GH26 | 15 | 5 | 18 | 369 | 28 | 42 | | 3 | | 6 | | 5 | 4 | 5 | 4 | | 8 | |
| GH28 | 6 | 2 | 18 | 472 | 50 | 18 | | 15 | | 9 | | 5 | 17 | 9 | 15 | | 14 | |
| Total | 86 | 19 | 80 | 2360 | 173 | 121 | | 29 | | 31 | | 18 | 38 | 19 | 24 | | 57 | |
| **Debranching enzymes** | | | | | | | | | | | | | | | | | | | |
| GH62 | 0 | 0 | 0 | 1 | 5 | 0 | | 0 | | 0 | | 0 | 0 | 0 | 1 | | 0 | |
| GH67 | 10 | 10 | 0 | 120 | 10 | 0 | | 2 | | 1 | | 2 | 3 | 2 | 2 | | 5 | |
| GH78 | 0 | 0 | 134 | 1260 | 67 | 48 | | 16 | | 9 | | 7 | 34 | 4 | 14 | | 38 | |
| Total | 10 | 10 | 134 | 1381 | 82 | 48 | | 18 | | 10 | | 9 | 37 | 6 | 17 | | 43 | |
| **Oligosaccharide degrading enzymes** | | | | | | | | | | | | | | | | | | | |
| GH1 | 22 | 61 | 38 | 253 | 105 | 93 | | 22 | | 22 | | 7 | 25 | 8 | 17 | | 25 | |
| GH2 | 23 | 24 | 745 | 1436 | 56 | 22 | | 22 | | 6 | | 5 | 28 | 11 | 12 | | 57 | |
| GH3 | 69 | 72 | 704 | 2844 | 328 | 273 | | 96 | | 74 | | 57 | 86 | 50 | 33 | | 69 | |
| GH29 | 0 | 2 | 110 | 939 | 133 | 40 | | 38 | | 27 | | 13 | 18 | 15 | 12 | | 25 | |
| GH35 | 3 | 3 | 48 | 158 | 22 | 11 | | 2 | | 2 | | 11 | 8 | 7 | 0 | | 11 | |
| GH38 | 11 | 3 | 68 | 272 | 44 | 49 | | 17 | | 12 | | 9 | 17 | 16 | 13 | | 32 | |
| GH39 | 3 | 1 | 9 | 315 | 67 | 68 | | 16 | | 30 | | 0 | 14 | 16 | 16 | | 27 | |
| GH42 | 24 | 8 | 45 | 374 | 38 | 37 | | 5 | | 4 | | 1 | 12 | 3 | 6 | | 18 | |
| Total | 155 | 174 | 1767 | 6591 | 793 | 593 | | 218 | | 177 | | 103 | 208 | 126 | 109 | | 264 | |
| Total GHs | 703 | 557 | 3828 | 27755 | 1280 | 896 | | 311 | | 259 | | 152 | 321 | 180 | 168 | | 439 | |

1. Quantification of 46 major CAG families detected based on ORFs containing the particular CAG domain. CAG families are sorted descending according to their average relative abundance across sludge samples.

| **CAG Family** | **Stanley_AS** | **Stanley_BF** | **ST_AS**  **summer** | **ST_AS**  **winter** | **ST_ADS** | **SWH_ADS** | **MAD** | **TAD** | **TCF** |
| --- | --- | --- | --- | --- | --- | --- | --- | --- | --- |
| **CE1** | 1449 | 1314 | 300 | 273 | 120 | 162 | 99 | 85 | 115 |
| **CBM50** | 615 | 633 | 240 | 239 | 135 | 193 | 120 | 154 | 232 |
| **CBM44^*^** | 798 | 277 | 317 | 199 | 191 | 262 | 78 | 58 | 179 |
| **GH109** | 1122 | 852 | 173 | 158 | 72 | 159 | 79 | 165 | 184 |
| **GH13** | 695 | 592 | 173 | 211 | 102 | 128 | 105 | 86 | 106 |
| **GH74^*^** | 912 | 512 | 419 | 314 | 72 | 71 | 53 | 22 | 95 |
| **CE10** | 824 | 945 | 147 | 131 | 68 | 101 | 58 | 66 | 60 |
| **GH23** | 552 | 694 | 153 | 131 | 68 | 85 | 65 | 41 | 68 |
| **CE4** | 562 | 665 | 108 | 87 | 63 | 101 | 44 | 61 | 66 |
| **GH3^*^** | 328 | 273 | 96 | 74 | 57 | 86 | 50 | 33 | 69 |
| **CBM48** | 367 | 280 | 78 | 98 | 43 | 61 | 47 | 35 | 30 |
| **CBM32** | 311 | 155 | 72 | 71 | 49 | 60 | 32 | 25 | 35 |
| **CE14** | 285 | 271 | 77 | 71 | 26 | 35 | 33 | 32 | 44 |
| **AA6** | 133 | 157 | 34 | 28 | 73 | 51 | 28 | 31 | 70 |
| **CBM66** | 232 | 204 | 42 | 45 | 20 | 25 | 27 | 43 | 39 |
| **SLH^*^** | 11 | 6 | 1 | 1 | 8 | 134 | 13 | 47 | 94 |
| **CE3** | 253 | 231 | 52 | 45 | 22 | 35 | 19 | 10 | 25 |
| **CBM9** | 160 | 143 | 54 | 49 | 21 | 32 | 15 | 12 | 51 |
| **GH43** | 160 | 45 | 46 | 35 | 27 | 41 | 22 | 14 | 58 |
| **Dockerin^*^** | 121 | 85 | 15 | 29 | 34 | 41 | 5 | 37 | 57 |
| **GH5^*^** | 196 | 119 | 29 | 28 | 15 | 28 | 18 | 18 | 47 |
| **CBM40** | 171 | 88 | 37 | 32 | 14 | 19 | 16 | 26 | 35 |
| **GH33** | 160 | 118 | 46 | 55 | 4 | 15 | 12 | 27 | 27 |
| **GH16** | 126 | 65 | 34 | 38 | 11 | 32 | 27 | 6 | 21 |
| **PL22** | 167 | 103 | 24 | 30 | 30 | 14 | 10 | 6 | 28 |
| **CBM67** | 157 | 82 | 28 | 24 | 6 | 28 | 3 | 30 | 26 |
| **CE7** | 139 | 131 | 13 | 25 | 11 | 11 | 11 | 20 | 28 |
| **CE11** | 112 | 115 | 33 | 34 | 20 | 10 | 11 | 9 | 15 |
| **GH57** | 60 | 72 | 14 | 11 | 20 | 41 | 11 | 12 | 34 |
| **PL9** | 67 | 55 | 20 | 16 | 33 | 11 | 19 | 10 | 24 |
| **GH31** | 90 | 64 | 23 | 17 | 12 | 23 | 14 | 14 | 32 |
| **CBM37** | 97 | 33 | 43 | 34 | 16 | 18 | 14 | 7 | 16 |
| **GH4** | 68 | 62 | 14 | 20 | 4 | 14 | 15 | 24 | 36 |
| **GH130** | 49 | 34 | 13 | 14 | 10 | 19 | 26 | 11 | 39 |
| **GH2^*^** | 56 | 22 | 22 | 6 | 5 | 28 | 11 | 12 | 57 |
| **GH15** | 114 | 174 | 15 | 15 | 12 | 17 | 8 | 9 | 12 |
| **CBM13** | 97 | 42 | 13 | 15 | 4 | 98 | 2 | 0 | 7 |
| **GH73** | 78 | 76 | 31 | 15 | 22 | 10 | 17 | 6 | 6 |
| **Cohesin^*^** | 64 | 42 | 22 | 10 | 12 | 37 | 5 | 7 | 36 |
| **GH78** | 67 | 48 | 16 | 9 | 7 | 34 | 4 | 14 | 38 |
| **GH38** | 44 | 49 | 17 | 12 | 9 | 17 | 16 | 13 | 32 |
| **GH18** | 69 | 41 | 15 | 13 | 7 | 22 | 14 | 18 | 20 |
| **GH103** | 106 | 173 | 20 | 24 | 9 | 4 | 5 | 1 | 4 |
| **CBM56** | 38 | 11 | 7 | 3 | 4 | 7 | 50 | 1 | 6 |
| **AA7** | 104 | 142 | 11 | 17 | 4 | 5 | 7 | 6 | 13 |
| **AA3^*^** | 117 | 130 | 12 | 8 | 2 | 6 | 3 | 5 | 6 |

*: CAG families associated with cellulose hydrolysis.

1. Quantification of 40 major orders within the compared sludge samples based on 16S rRNA gene sequences. Orders are sorted descending according to their average relative abundance across sludge samples.

| Order | Stanley_AS | Stanley_BF | ST_AS  summer | ST_AS  winter | ST_ADS | SWH_ADS | MAD | TAD | TCF |
| --- | --- | --- | --- | --- | --- | --- | --- | --- | --- |
| Actinomycetales | 31.7% | 34.5% | 14.7% | 20.6% | 10.6% | 13.8% | 9.5% | 9.2% | 0.3% |
| Clostridiales | 0.8% | 0.2% | 1.7% | 5.7% | 13.9% | 19.6% | 18.7% | 32.4% | 21.3% |
| Thermotogales | 0.0% | 0.0% | 0.0% | 0.4% | 17.6% | 19.5% | 8.5% | 5.1% | 20.5% |
| Sphingobacteriales | 6.0% | 0.2% | 13.7% | 8.5% | 1.7% | 4.8% | 2.5% | 1.9% | 8.4% |
| Planctomycetales | 3.9% | 9.8% | 6.0% | 5.0% | 1.1% | 1.3% | 3.2% | 12.1% | 0.4% |
| Bacteroidales | 0.3% | 0.0% | 0.6% | 0.6% | 16.2% | 8.1% | 10.6% | 0.4% | 0.1% |
| Spirochaetales | 0.5% | 0.1% | 0.7% | 0.4% | 4.3% | 4.4% | 5.4% | 0.0% | 13.4% |
| Chloroflexales | 11.6% | 15.2% | 0.0% | 0.1% | 0.0% | 0.9% | 0.0% | 0.3% | 0.0% |
| Rhizobiales | 5.1% | 7.0% | 2.9% | 5.7% | 1.1% | 1.3% | 1.9% | 2.2% | 0.1% |
| Flavobacteriales | 2.3% | 0.1% | 12.2% | 7.4% | 1.2% | 0.9% | 1.0% | 0.3% | 0.0% |
| Rhodobacterales | 2.0% | 0.6% | 5.1% | 9.9% | 2.0% | 0.4% | 1.5% | 2.4% | 0.0% |
| Burkholderiales | 7.8% | 3.2% | 1.7% | 1.7% | 1.0% | 3.7% | 1.2% | 1.2% | 0.0% |
| Myxococcales | 5.0% | 2.1% | 5.9% | 3.7% | 0.3% | 0.8% | 0.4% | 0.5% | 0.0% |
| Methanosarcinales | 0.0% | 0.0% | 0.0% | 0.2% | 2.0% | 4.1% | 1.0% | 0.4% | 9.1% |
| Methanobacteriales | 0.0% | 0.0% | 0.0% | 0.1% | 0.0% | 0.6% | 0.0% | 0.3% | 14.7% |
| Chlamydiales | 0.1% | 0.1% | 2.4% | 0.7% | 0.6% | 0.3% | 1.1% | 9.0% | 0.0% |
| Nitrospirales | 1.4% | 1.3% | 2.2% | 1.3% | 0.6% | 2.9% | 0.3% | 0.0% | 4.0% |
| Acidimicrobiales | 0.5% | 1.4% | 3.4% | 4.0% | 0.6% | 0.9% | 1.4% | 1.6% | 0.0% |
| Xanthomonadales | 1.5% | 5.1% | 2.0% | 2.6% | 0.2% | 0.7% | 1.0% | 0.5% | 0.0% |
| Rhodospirillales | 1.7% | 3.1% | 0.7% | 2.2% | 0.1% | 1.1% | 0.1% | 1.0% | 0.0% |
| Rhodocyclales | 1.7% | 0.2% | 1.0% | 0.4% | 3.2% | 0.6% | 0.7% | 1.4% | 0.4% |
| Phycisphaerales | 1.2% | 3.0% | 1.1% | 0.7% | 0.6% | 0.4% | 0.9% | 1.3% | 0.0% |
| Solirubrobacterales | 0.3% | 2.2% | 0.9% | 0.5% | 0.2% | 0.6% | 1.6% | 2.7% | 0.0% |
| Erysipelotrichales | 0.0% | 0.0% | 0.0% | 0.0% | 0.1% | 0.1% | 7.8% | 0.1% | 0.0% |
| Synergistales | 0.0% | 0.0% | 0.0% | 0.2% | 2.4% | 0.6% | 1.1% | 1.6% | 1.8% |
| Chlorobiales | 0.3% | 0.1% | 1.4% | 1.0% | 0.2% | 0.3% | 0.9% | 0.8% | 2.1% |
| Bdellovibrionales | 0.4% | 0.0% | 1.3% | 0.8% | 2.5% | 1.1% | 0.2% | 0.5% | 0.0% |
| Cytophagales | 1.8% | 0.1% | 2.2% | 1.0% | 0.3% | 0.3% | 0.5% | 0.5% | 0.0% |
| Verrucomicrobiales | 0.3% | 0.1% | 1.5% | 2.2% | 0.2% | 0.4% | 0.2% | 0.3% | 0.0% |
| Syntrophobacterales | 0.0% | 0.1% | 0.0% | 0.1% | 2.2% | 0.6% | 1.9% | 0.0% | 0.0% |
| Oceanospirillales | 0.0% | 0.1% | 2.2% | 1.3% | 0.0% | 0.1% | 0.3% | 0.6% | 0.0% |
| Lactobacillales | 0.5% | 0.4% | 0.5% | 0.7% | 1.1% | 0.1% | 0.3% | 0.8% | 0.0% |
| Bacillales | 0.1% | 0.3% | 0.3% | 0.2% | 0.1% | 0.2% | 1.7% | 1.1% | 0.2% |
| Deferribacterales | 0.1% | 0.0% | 2.0% | 0.7% | 0.1% | 0.5% | 0.3% | 0.1% | 0.0% |
| Sphingomonadales | 0.6% | 0.6% | 0.6% | 1.3% | 0.2% | 0.3% | 0.1% | 0.0% | 0.0% |
| Methanomicrobiales | 0.0% | 0.0% | 0.0% | 0.2% | 1.3% | 0.2% | 0.2% | 1.7% | 0.0% |
| Desulfuromonadales | 0.0% | 0.3% | 1.4% | 1.3% | 0.1% | 0.0% | 0.0% | 0.0% | 0.0% |
| Desulfobacterales | 0.0% | 0.0% | 0.0% | 0.1% | 1.9% | 0.0% | 0.3% | 0.0% | 0.0% |
| Neisseriales | 1.3% | 0.2% | 0.1% | 0.2% | 0.2% | 0.1% | 0.0% | 0.0% | 0.0% |
| Acholeplasmatales | 0.0% | 0.0% | 0.1% | 0.0% | 0.2% | 0.0% | 1.3% | 0.1% | 0.0% |

1. Topological properties of the co-occurrence network (positive network) of 46 major CAG families and 40 prevalent phylogenetic orders

| Node name | Module NO.^1)^ | Degree | Phylum affiliation |
| --- | --- | --- | --- |
| Acidimicrobiales | 12 | 1 | Actinobacteria |
| Oceanospirillales | 12 | 1 | Proteobacteria |
| CBM37 | 11 | 2 | NA |
| Flavobacteriales | 11 | 2 | Bacteroidetes |
| Cytophagales | 11 | 2 | Deferribacteres |
| Deferribacterales | 11 | 2 | Bacteroidetes |
| Rhodobacterales | 11 | 1 | Proteobacteria |
| Verrucomicrobiales | 11 | 1 | Chlamydiae |
| Planctomycetales | 10 | 2 | Planctomycetes |
| Phycisphaerales | 10 | 2 | Planctomycetes |
| Solirubrobacterales | 10 | 2 | Actinobacteria |
| Bacillales | 10 | 1 | Firmicutes |
| GH43 | 9 | 1 | NA |
| Spirochaetales | 9 | 1 | Spirochaetes |
| Sphingobacteriales | 8 | 1 | Bacteroidetes |
| Chlorobiales | 8 | 1 | Bacteroidetes |
| Xanthomonadales | 7 | 9 | Proteobacteria |
| CE1 | 7 | 8 | Actinobacteria |
| Actinomycetales | 7 | 8 | NA |
| Sphingomonadales | 7 | 8 | Proteobacteria |
| GH103 | 7 | 7 | NA |
| Rhizobiales | 7 | 6 | Proteobacteria |
| Rhodospirillales | 7 | 5 | Proteobacteria |
| CE10 | 7 | 4 | NA |
| CE3 | 7 | 4 | NA |
| GH23 | 7 | 3 | Proteobacteria |
| Burkholderiales | 7 | 3 | NA |
| CE14 | 7 | 2 | NA |
| GH74 | 7 | 2 | NA |
| Myxococcales | 7 | 2 | Proteobacteria |
| CBM13 | 7 | 1 | NA |
| Desulfuromonadales | 7 | 1 | Proteobacteria |
| CBM67 | 6 | 1 | NA |
| GH78 | 6 | 1 | NA |
| SLH | 5 | 5 | NA |
| GH57 | 5 | 4 | NA |
| Thermotogales | 5 | 4 | Euryarchaeota |
| Synergistales | 5 | 4 | Synergistetes |
| Methanosarcinales | 5 | 4 | Thermotogae |
| AA6 | 5 | 2 | NA |
| CBM50 | 4 | 5 | Firmicutes |
| Clostridiales | 4 | 4 | NA |
| GH31 | 4 | 4 | NA |
| GH38 | 4 | 3 | NA |
| GH130 | 4 | 3 | NA |
| GH2 | 4 | 2 | NA |
| CBM48 | 3 | 1 | NA |
| GH13 | 3 | 1 | NA |
| CE7 | 2 | 1 | NA |
| CBM66 | 2 | 1 | NA |
| GH109 | 1 | 2 | NA |
| CE4 | 1 | 1 | NA |
| GH15 | 1 | 1 | NA |

1): network modules determined by the Louvain algorithm

NA: not applicable

1. Observed and random co-occurring incidence within network modules.

| Module NO. | Observed incidence | Random incidence |
| --- | --- | --- |
| 1 | 0.0% | 0.2% |
| 2 | 0.0% | 0.1% |
| 3 | 0.0% | 0.1% |
| 4* | 1.3% | 1.1% |
| 5* | 1.3% | 1.1% |
| 6 | 0.0% | 0.1% |
| 7* | 9.3% | 8.7% |
| 8 | 0.0% | 0.1% |
| 9 | 0.0% | 0.1% |
| 10 | 0.0% | 0.4% |
| 11* | 1.3% | 1.1% |
| 12 | 0.0% | 0.1% |

*: modules whose nodes showed higher connectivity than random association.

1. Information of the metagenomic libraries of sludge samples and technical/biological replicates.

| Microbiome | Data size | DNA concentration (ng/µl) | DNA 260/280 | MG-RAST accession |
| --- | --- | --- | --- | --- |
| Stanley_AS | 17.9G | 48.9 | 1.78 | 4518784.3; 4518783.3 |
| Stanley_BF | 18.7G | 72.6 | 1.87 | 4518786.3; 4518785.3 |
| ST_AS_winter | 5.1G | 120.1 | 1.88 | 4518787.3；4489146.3 |
| ST_AS_summer | 5.1G | 128.8 | 1.92 | 4489104.3；4489073.3 |
| ST_ADS | 3.3G | 120.5 | 1.70 | 4504598.3；4504600.3 |
| SWH_ADS | 3.8G | 152.7 | 1.68 | 4504632.3；4504638.3 |
| MAD | 2.7G | 78 | 1.79 | 4485328.3；4485789.3 |
| TAD | 2.4G | 53 | 1.76 | 4486074.3；4488559.3 |
| TCF | 6.0G | 209.0 | 1.88 | 4503055.3; 4503056.3 |
| ADS_ ST_2011-9* | 1.2G | 74.2 | 1.80 | 4485272.3; 4485273.3 |
| ADS_ SWH_2011-9* | 1.2G | 62.9 | 1.80 | 4485327.3; 4485326.3 |
| ADS_ SWH_2012-3_1* | 4.2G | 147.6 | 1.68 | 4504628.3; 4504630.3 |

*: sludge samples used as the technical or biological replicates as illustrated in Figure S1

1. Statistics of assembled scaffolds from metagenome of sludge samples and technical/biological replicates.

| Microbiome | Total Base Mb | Number of Scaffolds | N50 bp | N90 bp | Largest Scaffold bp | ORFs number | Full-length ORFs percentage |
| --- | --- | --- | --- | --- | --- | --- | --- |
| Stanley_AS | 584 | 893,717 | 670 | 343 | 220,560 | 1,217,440 | 11.2% |
| Stanley_BF | 610 | 1,005,078 | 595 | 336 | 66,462 | 1,348,161 | 9.4% |
| ST_AS_winter | 135 | 469,046 | 558 | 332 | 61,203 | 300,883 | 8.2% |
| ST_AS_summer | 141 | 473,756 | 581 | 333 | 150,666 | 306,862 | 8.8% |
| ST_ADS | 76 | 119,033 | 639 | 340 | 161,411 | 158,417 | 11.6% |
| SWH_ADS | 98 | 132,597 | 821 | 349 | 80,491 | 187,835 | 16.6% |
| MAD | 62 | 189,686 | 660 | 342 | 46,325 | 128,509 | 11.9% |
| TAD | 64 | 159,860 | 956 | 359 | 120,423 | 116,511 | 17.5% |
| TCF | 76.9 | 27,384 | 10,963 | 976 | 331,825 | 91,808 | 61.6% |
| ADS_ ST_2011-9* | 24 | 77,066 | 618 | 340 | 43,565 | 50,448 | 9.8% |
| ADS_ SWH_2011-9* | 34 | 99,050 | 739 | 351 | 20,480 | 67,636 | 11.7% |
| ADS_ SWH_2012-3_1* | 106 | 142,673 | 833 | 350 | 86,769 | 203,484 | 17.0% |

*: sludge samples used as the technical or biological replicates as illustrated in Figure S1

1. Illustration of the experimental design of metagenomes used for this study. Frames of technical and biological replicates are respectively filled with blue and green color.
2. Rarefaction analysis of the sludge metagenomes
3.
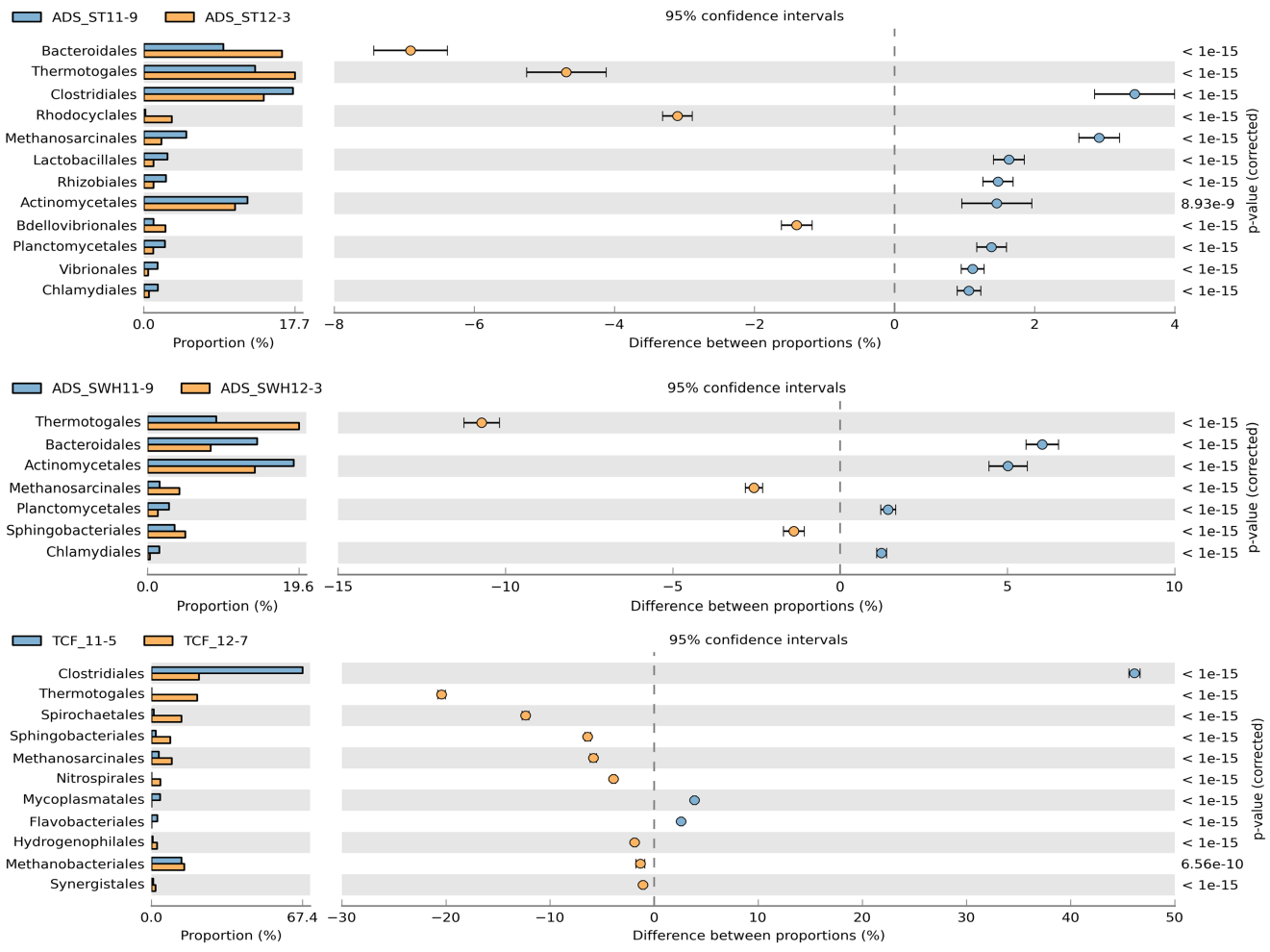
Phylogenetic orders showed significant variation (P-value< 0.05 and proportion difference > 1%) between biological replicates.
4. Similarity distribution of GH-encoding ORFs to their best BLASTN hit against NCBI *nr* database. Left and right figure respectively shows the ORFs counts and accumulative abundance of GH-encoding ORFs from different sludge microbiomes.

1. Heatmap of the most prevalent phylogenetic groups (order level) determined by the CAGs-encoding genes (a) and 16S rRNA gene sequences (b).
2. Major CAG families showed significant variation (p-value < 0.05 by one-way ANOVA analysis) among sludge samples with different dissolved oxygen (left), temperature (middle) and salinity (right). Abbreviations in the figures: M: Mesophilic; A: Ambient temperature; T: Thermophilic.
3. Whole network among 46 major CAG families and 40 prevalent phylogenetic orders. Nodes representing either CAG families or phylogenetic orders, are colored according to the network modules (that is clusters) determined by multi-level aggregation method (Louvain algorithm (Blondel et al., 2011)). Each edge represents a strong (Spearman's rank correlation coefficient r^2^ > 0.6) and significant (p-value < 0.01) correlation between node-pairs. Edges are colored according to the value of r^2^ with red stands for positive correlation; blue represents negative correlation. The size of each node and the font size of label is proportion to the number of connections (that is degree) of that node. And the thickness of edge is proportion to the correlation coefficient between nodes.
4. Co-exclusion network (that is the negative network) among 46 major CAG families and 40 prevalent phylogenetic orders. Nodes representing either CAG families or phylogenetic orders, are colored according to the network modules (that is clusters) determined by multi-level aggregation method (Louvain algorithm (Blondel et al., 2011)). Each edge representing a strong (Spearman's rank correlation coefficient r^2^ > 0.6) and significant (p-value < 0.01) correlation between node-pairs, are in the same color with its source node. The size of each node and the font size of label is proportion to the number of connections (that is degree) of that node. And the thickness of edge is proportion to the correlation coefficient between nodes.
